# Tuberculosis Biomarker Extraction and Isothermal Amplification in an Integrated Diagnostic Device

**DOI:** 10.1371/journal.pone.0130260

**Published:** 2015-07-01

**Authors:** Amy Creecy, Patricia K. Russ, Francesca Solinas, David W. Wright, Frederick R. Haselton

**Affiliations:** 1 Department of Biomedical Engineering, Vanderbilt University, Nashville, TN, United States of America; 2 Department of Chemistry, Vanderbilt University, Nashville, TN, United States of America; Universidade Nova de Lisboa, PORTUGAL

## Abstract

In this study, we integrated magnetic bead-based sample preparation and isothermal loop mediated amplification (LAMP) of TB in a single tube. Surrogate sputum samples produced by the Program for Appropriate Technology in Health containing inactivated TB bacteria were used to test the diagnostic. In order to test the sample preparation method, samples were lysed, and DNA was manually extracted and eluted into water in the tube. In a thermal cycler, LAMP amplified TB DNA from 10^3^ TB cells/mL of sputum at 53.5 ± 3.3 minutes, 10^4^ cells/mL at 46.3 ± 2.2 minutes, and 10^5^ cells/mL at 41.6 ± 1.9 minutes. Negative control samples did not amplify. Next, sample preparation was combined with in-tubing isothermal LAMP amplification by replacing the water elution chamber with a LAMP reaction chamber. In this intermediate configuration, LAMP amplified 10^3^ cells/mL at 74 ± 10 minutes, 10^4^ cells/mL at 60 ± 9 minutes, and 10^5^ TB cells/mL of sputum at 54 ± 9 minutes. Two of three negative controls did not amplify; one amplified at 100 minutes. In the semi-automated system, DNA was eluted directly into an isothermal reaction solution containing the faster OptiGene DNA polymerase. The low surrogate sputum concentration, 10^3^ TB cells/mL, amplified at 52.8 ± 3.3 minutes, 10^4^ cells/mL at 45.4 ± 11.3 minutes, and 10^5^ cells/mL at 31.8 ± 2.9 minutes. TB negative samples amplified at 66.4 ± 7.4 minutes. This study demonstrated the feasibility of a single tube design for integrating sample preparation and isothermal amplification, which with further development could be useful for point-of-care applications, particularly in a low-resource setting.

## Introduction

According to the World Health Organization (WHO), nine million individuals were infected with tuberculosis (TB) in 2014, and TB is second only to HIV in cause of death due to an infectious agent [1]. The highest rates of incidence occur in Africa and southeast Asia and are often coincident with low-resource areas of the world. While the spread of TB is declining, an untreated person with active TB infects an average of 10–15 people per year [2]. Particularly as drug-resistant strains of the pathogen emerge, continued improvements in diagnosis and treatment of TB are critical to controlling the spread of the disease and to efforts to eradicate it.

Detection and, therefore, treatment of tuberculosis is challenging in areas where the TB burden is often the highest. The standard method for diagnosis of active TB in low-resource areas is sputum smear microscopy [3]. However, sputum smear only detects the most infectious cases, with a limit of detection of 10^4^ mycobacterium/mL of sputum [4]. Accuracy is heavily dependent on the experience of the technician, and the technicians themselves are often at risk of exposure [5]. The reference standard for TB diagnosis is bacterial culture, which can be used to determine drug resistance, but it takes a minimum of one week to yield results [6]. Nucleic acid amplification tests are more sensitive than sputum smear, faster than bacterial culture, and can also be used to identify drug resistant strains, which are becoming increasingly prevalent [1]. However, nucleic acid amplification tests often require expensive equipment and trained personnel not available in low-resource areas.

Recent efforts have been directed towards development of technologies to deliver nucleic acid based TB diagnosis to areas with high disease burden. The WHO recommended Xpert MTB/RIF system (Cepheid) combines sample preparation and polymerase chain reaction (PCR) and has been employed in over 100 high burden countries [1], but its use is still limited by cost of support and maintenance [7]. Isothermal amplification of biomarker DNA has also been employed in TB diagnostic development, because it combines the sensitivity and specificity of nucleic acid based detection with simple instrument requirements. Isothermal Loop Mediated Amplification (LAMP) of TB DNA from clinical samples has been detected visually by turbidity [8] and fluorescence intercalating dye [9], by incorporation with a lateral flow dipstick [10], and by fluorescence detector [11]. Those targeting the IS6110 gene of *Mycobacterium tuberculosis* with LAMP report near 100% specificity [8,10,11].

The WHO also reports that the preparation of patient samples for nucleic acid amplification is another significant limitation to the utility of molecular technologies at the point of care [2]. There are some isothermal amplification based diagnostics available that incorporate sample preparation, but they have low sensitivity [12] or require approximately one hour of technician hands-on time [13]. In this study, we combine a self-contained, easy to use sample preparation technique [14] with isothermal amplification in order to detect the IS6110 gene of *M*. *tuberculosis* extracted from surrogate sputum samples as a potential low-resource diagnostic.

The first component of the integrated design is sample preparation, which is often required in order to remove inhibitors of amplification. We have developed a self-contained sample preparation technique that does not require the equipment and trained personnel of a laboratory-based assay [14–17]. Using our technique, nucleic acids in a sample are adsorbed to the surface of silica coated magnetic beads in binding buffer. Nucleic acid extraction is achieved by pulling the magnetic beads using external magnets through extraction solutions arrayed in plastic tubing separated by surface tension valves [14–17]. Nucleic acids are eluted in the final chamber of the tubing for amplification.

Next we add nucleic acid amplification and biomarker detection. LAMP relies on a strand displacing polymerase and method specific primer designs [18]. In LAMP, two sets of primers, an inner and an outer set, provide specificity to the target DNA sequence by binding six distinct sequences. As the outer primers are extended, they displace the extended inner primers, which are then able to self-anneal to produce a stem-loop structure. The stem-loop structures provide binding sites for additional primers resulting in exponential amplification of the target sequence at 65°C. As in PCR, amplification product can be detected by measuring fluorescence of a DNA intercalating dye.

In this study, we verify that TB DNA can be extracted from surrogate sputum samples using our self-contained sample preparation technique. We optimize LAMP amplification of the IS6110 gene of *Mycobacterium tuberculosis* for use in our design. Finally, the two techniques are integrated within a single tube and evaluated for TB detection from surrogate sputum samples.

## Materials and Methods

### Surrogate sputum samples

Surrogate sputum samples were generously provided by the Program for Appropriate Technology in Health (PATH), a nonprofit global health organization based in Seattle, in lieu of actual sputum samples. These samples contained artificial sputum composed of 47 mg/mL of Type II porcine mucin, 6 mg/mL of salmon sperm DNA, 3.6 mg/mL phosphatidylcholine, 33 mg/mL bovine serum albumin, 114 mM sodium chloride and 2 mM sodium azide. These concentrations are based on the component concentrations of sputum determined by Sanders et al [19]. Artificial sputum was mixed overnight with known amounts of chemically inactivated *Mycobacterium tuberculosis* (Rif sensitive, clone H37Rv Johannesburg) at 4°C to obtain a uniform slurry. Bacteria were previously chemically inactivated with SR Buffer (Cepheid) and provided to PATH from Drs. Wendy Stevens, Bavesh Kana and Lesley Scott at the University of Witwatersrand. Bacteria samples shipped to PATH exhibited no growth for 42 days. Bacteria were counted by a Guava Easycyte mini microcapillary flow cytometer after being gently rocked with 400 um glass beads to disperse large aggregates. Surrogate sputum samples were spiked with TB to produce three different concentrations: 10^3^ cells/mL (low), 10^4^ cells/mL (medium), and 10^5^ cells/mL (high). Surrogate sputum without bacteria was used as a negative sample, giving a total of four different concentrations.

### Chemical lysis of TB mycobacteria in surrogate sputum

**Fig 1:**
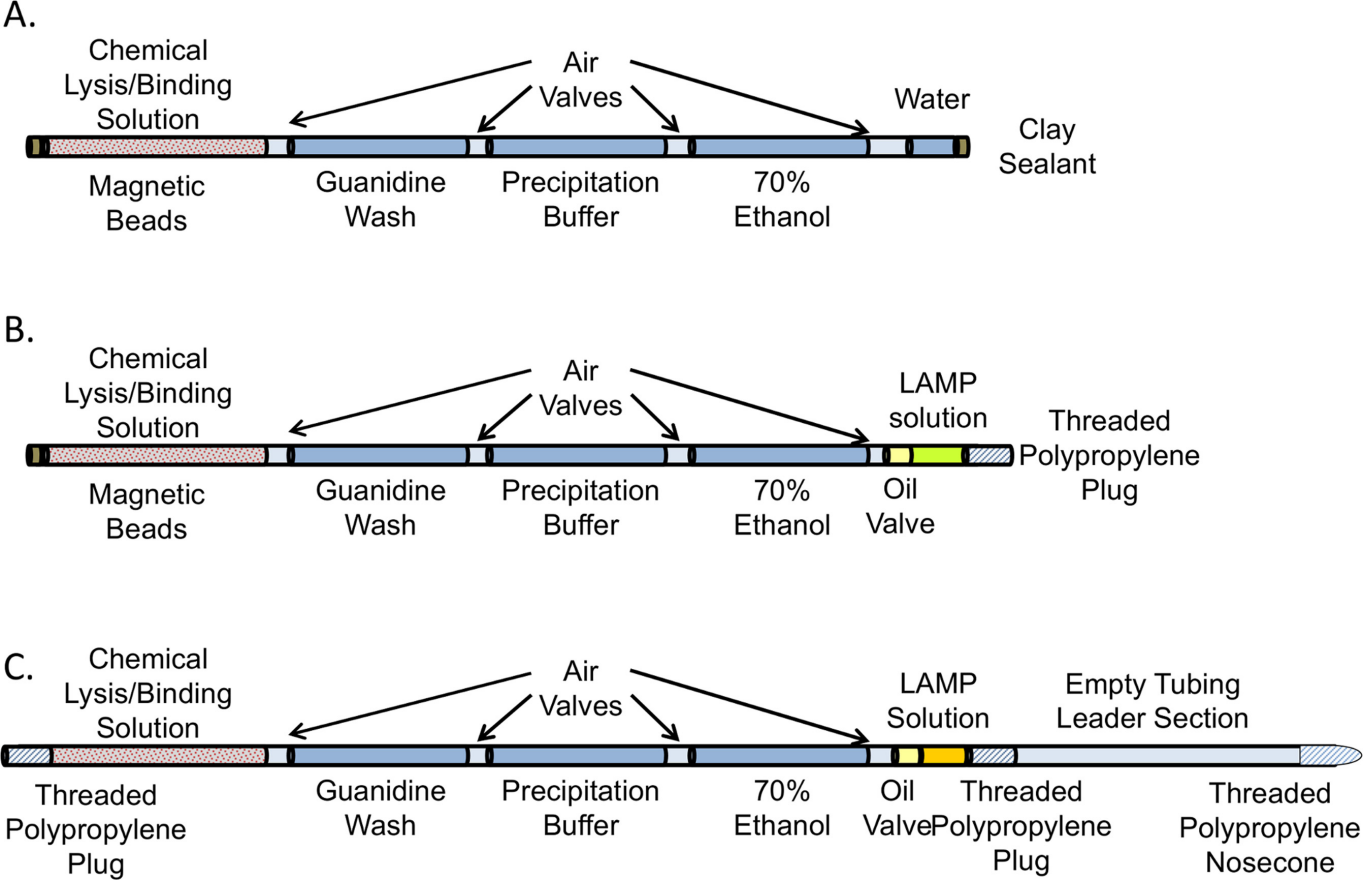
Low-resource DNA extraction technique. A. Lysed PATH samples were loaded into the extraction tubing as the first chamber. An external magnet was used to pull the binding beads through the solutions, and the DNA was eluted in the final chamber. B. Low-resource DNA extraction was combined with in-tube amplification. After the lysed sputum sample was introduced into the tubing, an oil valve was added to the opposite end to prevent evaporation of the LAMP reaction solution that followed. C. The tubing for automated DNA extraction and amplification is the same as the tubing in B with the addition of a leader section that guided the tubing through the instrument during the assay, as shown in Fig 2 and SI1.

Chemical lysis was performed to release the bacterial DNA into the sputum [20–22]. In 2 mL tubes, 500 uL of surrogate sputum were mixed with 500 uL of lysis and binding buffer (4M guanidine thiocyanate, 25 mM sodium citrate, 4.9% Triton X-100, 0.2% sodium dodecyl sulfate) and 0.8 mg of MyOne Silane Dynal beads. This mixture was agitated for 10 minutes on a Fisher Vortex Genie 2 at speed 4. After agitation, 1 mL of lysed sample was pipetted into the tubing for extraction (Fig 1). The procedure was repeated for each concentration of TB in surrogate sputum: 0, 10^3^, 10^4^, and 10^5^ cells/mL.

### Low-resource DNA extraction technique

An extraction technique [14] based on adsorption of DNA to silica coated magnetic beads was used to extract DNA from chemically lysed surrogate sputum samples. Extraction solutions were arrayed within fluorinated ethylene propylene (FEP) tubing with an inner diameter of 0.23 cm and an outer diameter of 0.31 cm (Fig 1A). Solutions were loaded by pipetting into one end of the tubing. Air valves held in place by surface tension forces separated the solutions. The elution chamber, containing 50 ul of water, was followed by 300 ul of 70% ethanol, 300 ul of precipitation solution (80% Ethanol, 5 mM potassium phosphate), and 300ul of wash solution (4M guanidine hydrochloride, 25 mM sodium citrate). Once the sputum sample was lysed, it was added to the tubing, and the ends of the FEP tubing were sealed.

In the manual version of the assay (Fig 1A and 1B), a permanent magnet was used to pull the DNA-bound magnetized beads from one solution to another. The beads were dispersed within each chamber of the extraction tubing by moving the magnet rapidly back and forth. Then the beads were collected at the edge of the chamber before being magnetically moved through the air valve into the next chamber. In the final chamber, DNA on the beads was released in the elution solution, and then the beads were pulled back into the previous chamber. The elution chamber was cut off, and the DNA was subsequently amplified by LAMP or PCR.

### LAMP and PCR Amplification

LAMP reactions were performed in a final volume of 50 ul at 65°C. The isothermal reaction consisted of 10 mM Tris HCl (pH 8.8), 10 mM KCl, 10 mM (NH_4_)_2_SO_4_, 1 mM MgSO_4_, 1 M Betaine, 0.6 mM dNTPs, 0.1% Tween 20, 12 units Bst 2.0 DNA polymerase, 1 uM SYTO-9, and primers (Table 1). LAMP primers were designed using PrimerExplorer v4, available online (http://primerexplorer.jp/e/). In the complete design, the reaction buffer and Bst 2.0 polymerase were replaced with OptiGene Isothermal Master Mix (OptiGene, United Kingdom) containing GspM2.0 polymerase used according to manufacturer’s instructions. SYTO-9 was replaced by 0.1 uM SYTO-82 (Life Technologies, NY).

**Table 1:** Primer Sequences.

	Primer	Sequence (5’ to 3’)	Concentration in Reaction (uM)
**LAMP**	F3	TGATCCGGCCACAGCC	0.2
	B3	TCGTGGAAGCGACCCG	0.2
	FIP	GCTACCCACAGCCGGTTAGGTGTCCCGCCGATCTCGT	1.6
	BIP	TCACCTATGTGTCGACCTGGGCGCCCAGGATCCTGCGA	1.6
**PCR**	Forward	ACCAGCACCTAACCGGCTGTGG	0.2
	Reverse	CATCGTGGAAGCGACCCGCCAG	0.2

In order to compare the reaction buffer and Bst 2.0 polymerase with the OptiGene Isothermal Master Mix and GspM2.0 polymerase, LAMP reactions were performed with the pGEM-T Easy Vector plasmid (Promega) with an insert of the IS6110 sequence (gift from USTAR). Five hundred, 5X10^4^, and 5X10^6^ copies per reaction as well as no template controls were compared. Reactions were performed in a Rotor-Gene Q thermal cycler (Qiagen) with the protocol described in Table 2.

**Table 2:** Rotor-Gene Q instrument set-up.

	PCR		LAMP	
Step	Temp	Time	Temp	Time
Hold	95°C	15 min	N/A	N/A
Cycles/Time		40 cycles		80 cycles
Melt	95°C	15 sec		
Anneal	62°C	30 sec		
Read	72°C	30 sec	65°C	1 min

PCR was performed in a final volume of 25 ul using primers (Table 1) from Cannas et al [5] and a QuantiTect SYBR Green PCR kit (Qiagen). PCR reactions were performed in a Rotor-Gene Q thermal cycler (Qiagen) with the protocol described in Table 2.

A fluorescence threshold was used to calculate the amplification time for each reaction. The threshold of 1.6 relative fluorescence units (RFU) was within the linear range of the amplification curves and above the background fluorescence seen with primers and no target DNA. To compare PCR and LAMP reaction times, the threshold cycle number (Ct) was converted to elapsed time. To calculate amplification time for PCR, Ct was multiplied by 85/60. This ratio was based on the 75 second duration of the cycle, plus 10 sec of ramp time between the three different temperatures, and dividing by 60 to convert to minutes. The 15 minute hold step at 95°C was then added. For LAMP, 80 cycles of 1 min at 65°C was programmed in the Rotor-Gene software. Amplification time was calculated by multiplying Ct by 85/75. This ratio accounted for the transition time between one minute holds in the Rotor-Gene program where the sample remained at the amplification temperature.

### Manual DNA extraction with in-tube amplification

Manual extraction of lysed surrogate sputum samples was performed as above with the following modifications. Twenty microliters of PCR grade mineral oil was added after the air valve before the elution chamber. The elution chamber was changed from water to 50 ul of LAMP reaction solution, and the clay sealant was replaced by a polypropylene plug that could withstand heating (Fig 1B). Following elution of extracted DNA into LAMP solution, the beads were pulled back into the previous chamber. The tubing was then mounted vertically on the hot side of a Peltier heater monitored by a thermal camera. Fluorescence was measured by an ESElog fluorescence detector (Qiagen), exciting at 470 nm and reading at 520 nm. Fluorescence measurements were recorded every 30 seconds for up to 100 minutes. Data were normalized by dividing by the average of the baseline measurements [16]. The threshold value was chosen to be within the linear region of the normalized amplification curves when plotted on a log scale [17]. The time at which the normalized fluorescence was first above 1.6 RFU was chosen as the amplification time.

### Automated DNA extraction and amplification

**Fig 2:**
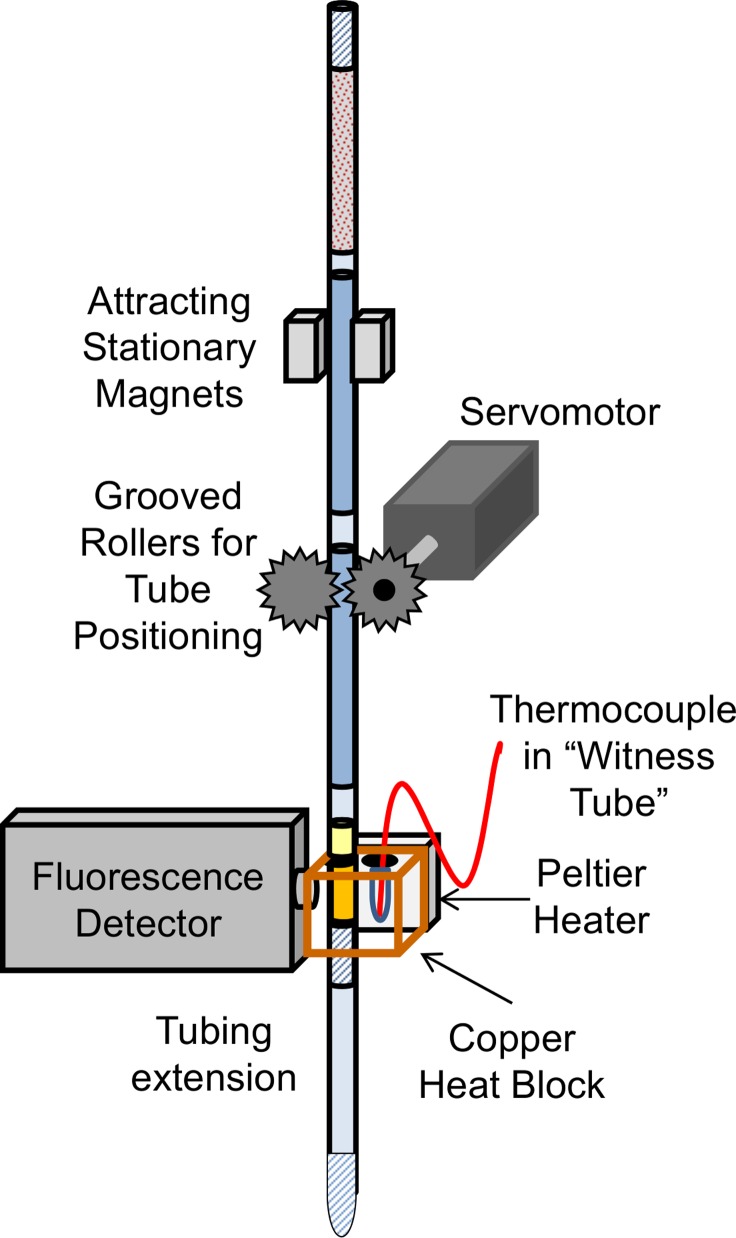
Integrated DNA extraction and amplification device. The extraction tubing was raised and lowered between attracting magnets to move the binding beads through the solutions into the isothermal reaction chamber. After the DNA eluted from the beads, the LAMP solution chamber was positioned for amplification in a copper heat block. The block held the reaction chamber at 65°C while the detector measured fluorescence over time. Diagram not to scale.

For automated DNA extraction and amplification, chemical lysis was performed on surrogate sputum samples provided by PATH. Tubing was loaded with lysed sample, buffers, and amplification reaction solution as described above (Fig 1C). The tubing was then loaded vertically into the automated extraction and amplification device (Fig 2). A servomotor (SGMAH-02B, Yaskawa America Inc., Waukegan, IL) controlled by LabView (National Insturments, Austin, TX) software moved the tubing during the assay. The shaft of the motor was fitted with a grooved roller to grip and position the FEP tubing during processing. Inclusion of a second grooved roller increased the surface area of the tubing in contact with the rollers, and coupling the second roller to the first by interlocking teeth improved tubing motion. As the motor turned, the tubing was raised and lowered between a pair of permanent magnets. The magnets were positioned to attract north-south across the tube. The velocity and position of the tube was used to position the magnetic binding beads within the appropriate processing chamber. The successive positions of the tube and the beads are shown in SI1. Briefly, the tubing was first positioned with the reaction chamber in the heat block in front of the fluorescence reader (P1). The tubing was then slowly lowered at 0.1 cm/sec past the magnets. At this speed the beads are held between the magnets, and the tube motion collects the beads at the back edge of the sample chamber (P2). The tubing was raised at the same rate in order to gather the beads at the front edge of the sample chamber and pull them across the air valve into the guanidine wash solution (P3). Then the tubing was moved upward at 5 cm/sec to a height 5cm above the magnets. At this faster speed the magnetic beads are pulled away from the magnets and are dispersed in the wash solution chamber (P4). The tubing was held stationary for 5 sec, and the beads slowly settled within the solution. Then the tubing was moved down at 5 cm/sec to reposition the chamber between the magnets (P5). The tubing was moved upwards at 0.1 cm/sec to pull the beads through the next air valve into the following chamber (P6). The pattern was repeated as the beads were transported down the tubing (P7–P11), finally arriving in the isothermal solution (P12). After the beads were dispersed within the isothermal solution (P13), the beads were withdrawn from the isothermal reaction, and the reaction chamber was moved to the heat block by slowly lowering the tubing at 0.1 cm/sec (P14).

For amplification, the isothermal solution chamber of the tubing was positioned in a copper heat block (Fig 2). The heat block was in contact with the hot side of a Peltier heater (VT-31-1.0–1.3, TE Technology, Traverse City, MI). The temperature was set to 65°C by adjusting the current prior to the start of the experiment. The heat block also contained a thermocouple in a “witness tube” used to monitor the reaction temperature. In addition, a hole was drilled at a right angle to the tubing to allow optical measurement of reaction fluorescence in the tubing. Fluorescence measurements were taken with an ESElog fluorescence detector (Qiagen), exciting with 520 nm and reading at 570 nm. Fluorescence measurements were recorded every 30 seconds for up to 100 minutes. Data were normalized by dividing by the average of the baseline measurements [16]. The threshold value was chosen to be within the linear region of the normalized amplification curves when plotted on a log scale [17]. The reading at which the normalized fluorescence was first above 1.6 RFU was chosen as the amplification time.

### Statistical analysis

Statistical significance between amplification times of different target concentrations was determined by ANOVA performed using SigmaPlot software. If a difference was detected among groups, all pairwise multiple comparison procedures (Holm-Sidak method) were performed. P < 0.05 was used to determine significant differences in amplification time.

## Results

### Amplification of TB DNA extracted from chemically lysed surrogate sputum samples

**Fig 3:**
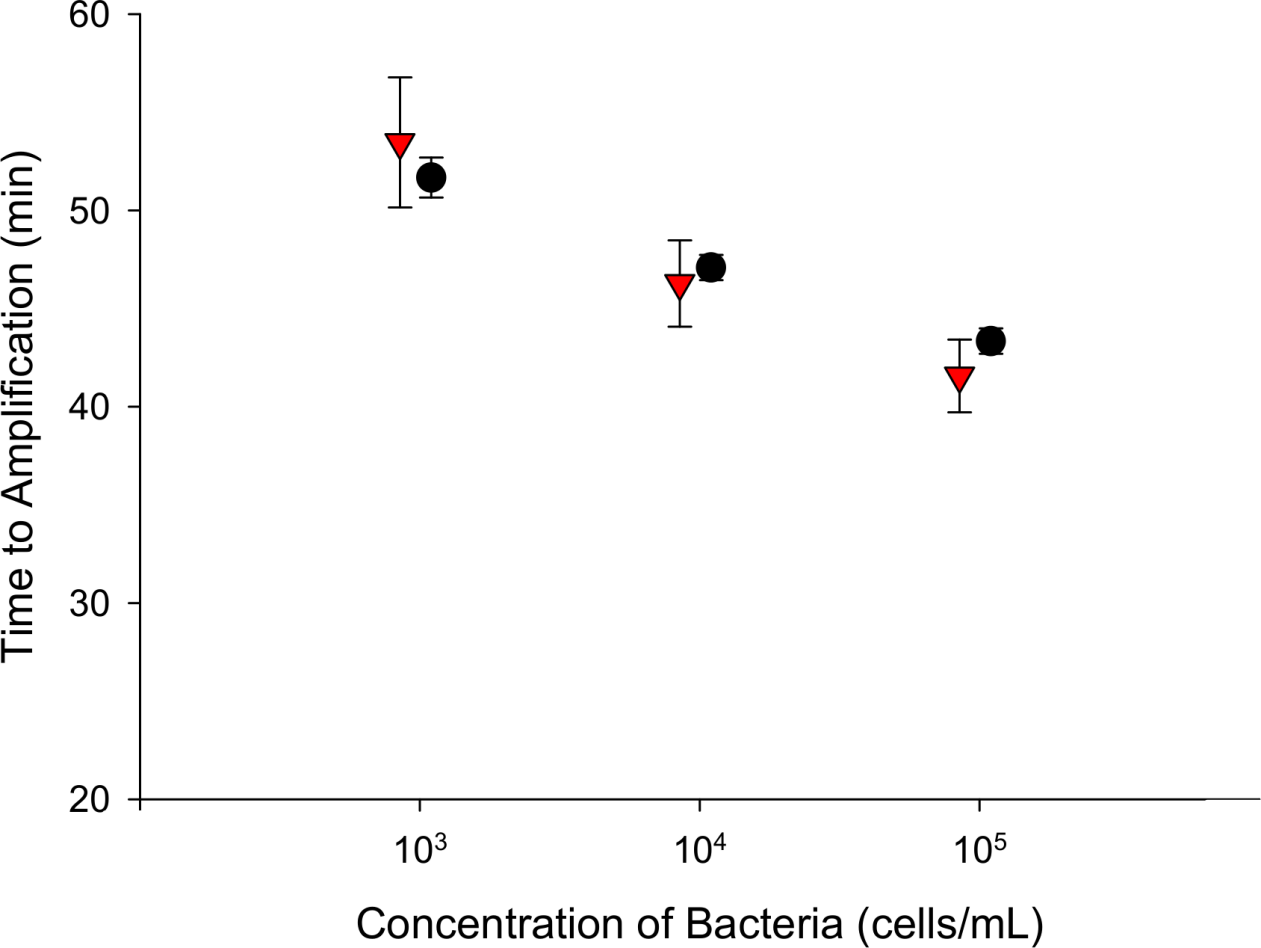
Time to amplification of TB DNA by LAMP and PCR. DNA was extracted from lysed surrogate sputum samples using the low-resource extraction technique and eluted into water. Eluent was amplified by LAMP (red triangle) and PCR (black circle); N = 6.

Surrogate sputum samples were chemically lysed, and DNA was manually extracted and eluted into water using the tube configuration shown in Fig 1A. The eluent was subsequently removed from the tubing and amplified by LAMP and PCR in a Rotor-Gene Q thermal cycler. PCR and LAMP amplified each of the three cell concentrations in similar reaction times (Fig 3). PCR amplified low (10^3^ cells/mL), medium (10^4^ cells/mL), and high (10^5^ TB cells/mL of sputum) samples at 51.7 ± 1.0, 47.1 ± 0.6, and at 43.3 ± 0.6 minutes respectively (N = 6). LAMP amplified low, medium, and high samples at 53.5 ± 3.3, 46.3 ± 2.2, and 41.6 ± 1.9 minutes (N = 6). Neither LAMP nor PCR amplified any of the negative samples before 90 min.

### Manual DNA extraction combined with in-tube amplification

**Fig 4:**
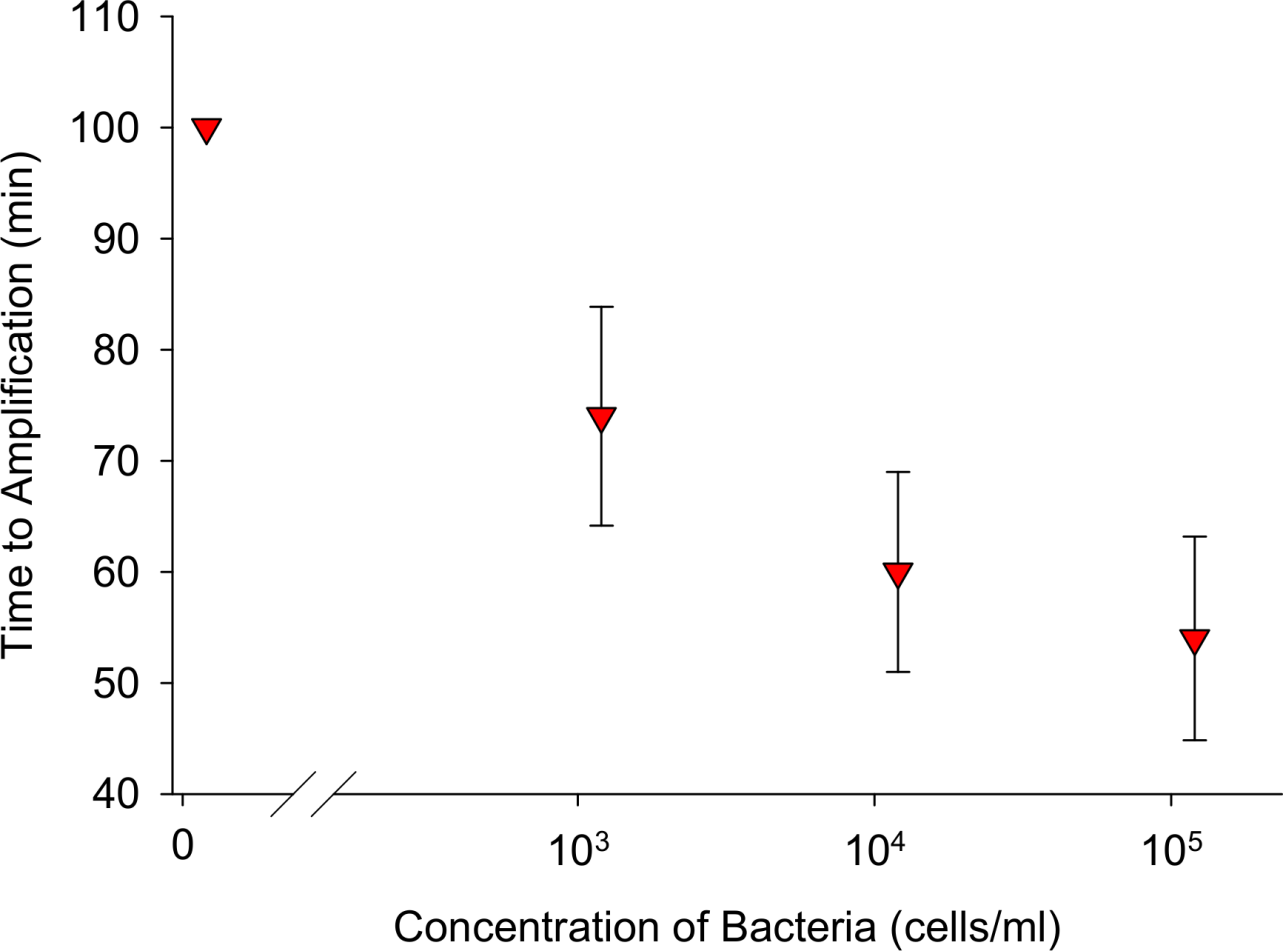
Time to amplification of TB DNA eluted directly into LAMP reaction solution. DNA was extracted from lysed surrogate sputum samples using the manual extraction technique and eluted into LAMP reaction solution. All three concentrations of surrogate sputum amplified in the extraction tubing. One negative control sample amplified in the 100 minute assay time. N = 3.

Surrogate sputum samples were chemically lysed, and DNA was manually extracted and eluted into LAMP reaction solution (Fig 1B). The tubing was mounted vertically with the reaction chamber in contact with the hot side of a Peltier heater maintained at 65°C for LAMP amplification. All three concentrations of surrogate sputum containing TB bacteria amplified (Fig 4). Two of three negative controls did not amplify; one amplified at 100 minutes. LAMP amplified 10^3^ cells/mL at 74 ± 10 min, 10^4^ cells/mL at 60 ± 9 min, and 10^5^ TB cells/mL of sputum at 54 ± 9 min (N = 3).

### Comparison of Bst to GspM2.0 polymerase in LAMP reactions

**Fig 5:**
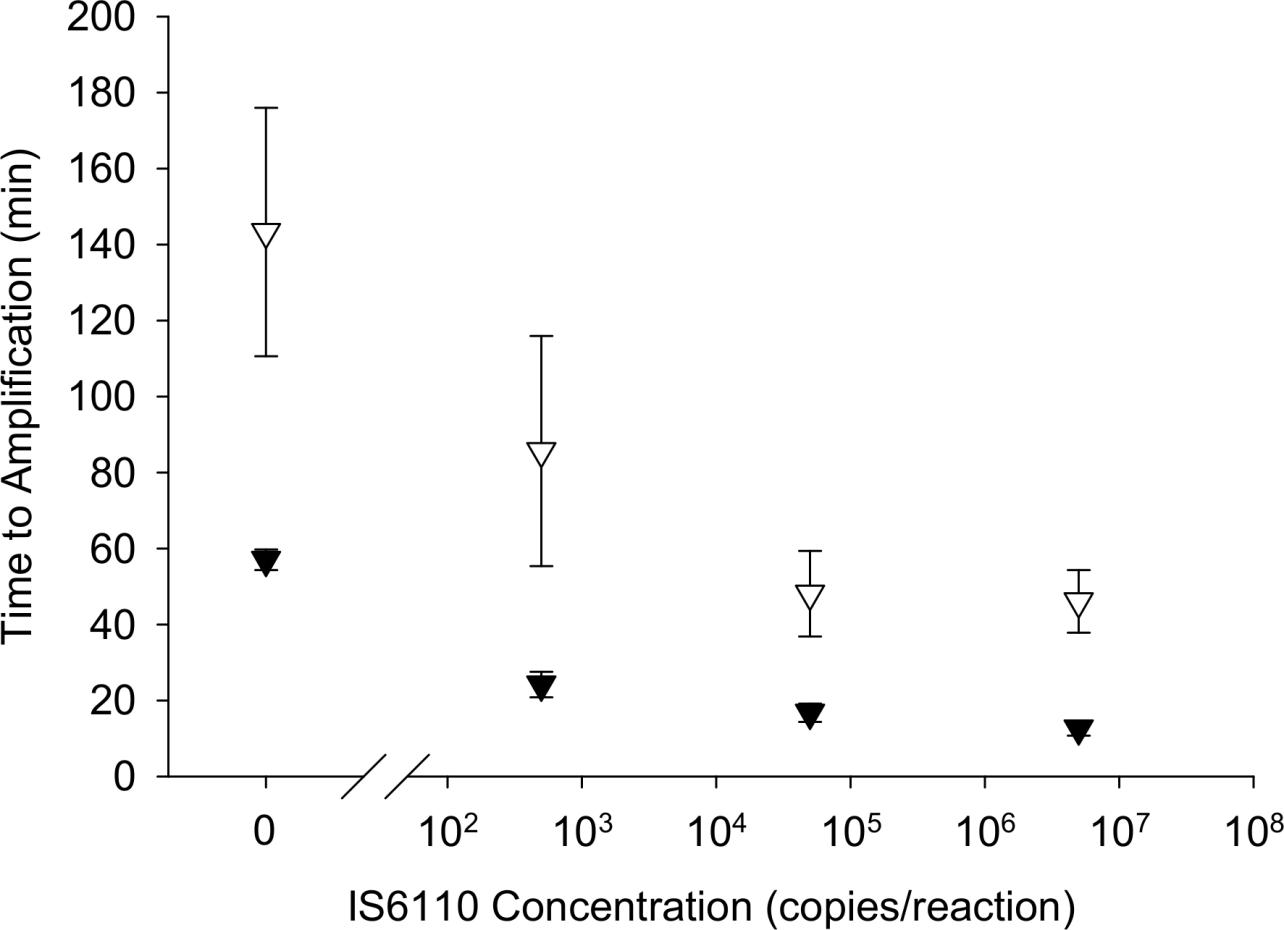
Comparison of Bst 2.0 and GspM2.0 polymerases in LAMP reactions. LAMP reactions were performed with plasmid DNA with an insert of the IS6110 sequence. In a Rotor-Gene Q thermal cycler, 5X10^2^, 5X10^4^, and 5X10^6^ copies per reaction as well as no template controls were compared. N = 4.

One of the goals of the integrated device design was to shorten the overall time to result. A significant portion of the total assay time is isothermal amplification time. In order to decrease amplification time we explored the use of a faster polymerase. We compared the Bst 2.0 polymerase and buffer to the OptiGene Master Mix containing GspM 2.0 polymerase in a Rotor-Gene Q thermal cycler using reactions spiked with plasmid DNA containing the IS6110 gene. Amplification times of all target concentrations tested were significantly shorter with the GspM 2.0 enzyme (Fig 5). With GspM 2.0, 5X10^2^ copies/reaction amplified in 21 ± 3 minutes, 5X10^4^ in 17 ± 2 minutes, and 5X10^6^ in 13 ± 2 minutes. With Bst 2.0, 5X10^2^ copies/reaction amplified in 86 ± 30 minutes, 5X10^4^ in 48 ± 11 minutes, and 5X10^6^ in 46 ± 8 minutes. The GspM 2.0 no template control time (57 ± 3 minutes) was also significantly shorter than the Bst 2.0 no template control (143 ± 33 minutes). However, the GspM 2.0 amplification times were all still significantly lower than the no template control.

### Integrated extraction and fast isothermal amplification of TB DNA from surrogate sputum samples

**Fig 6:**
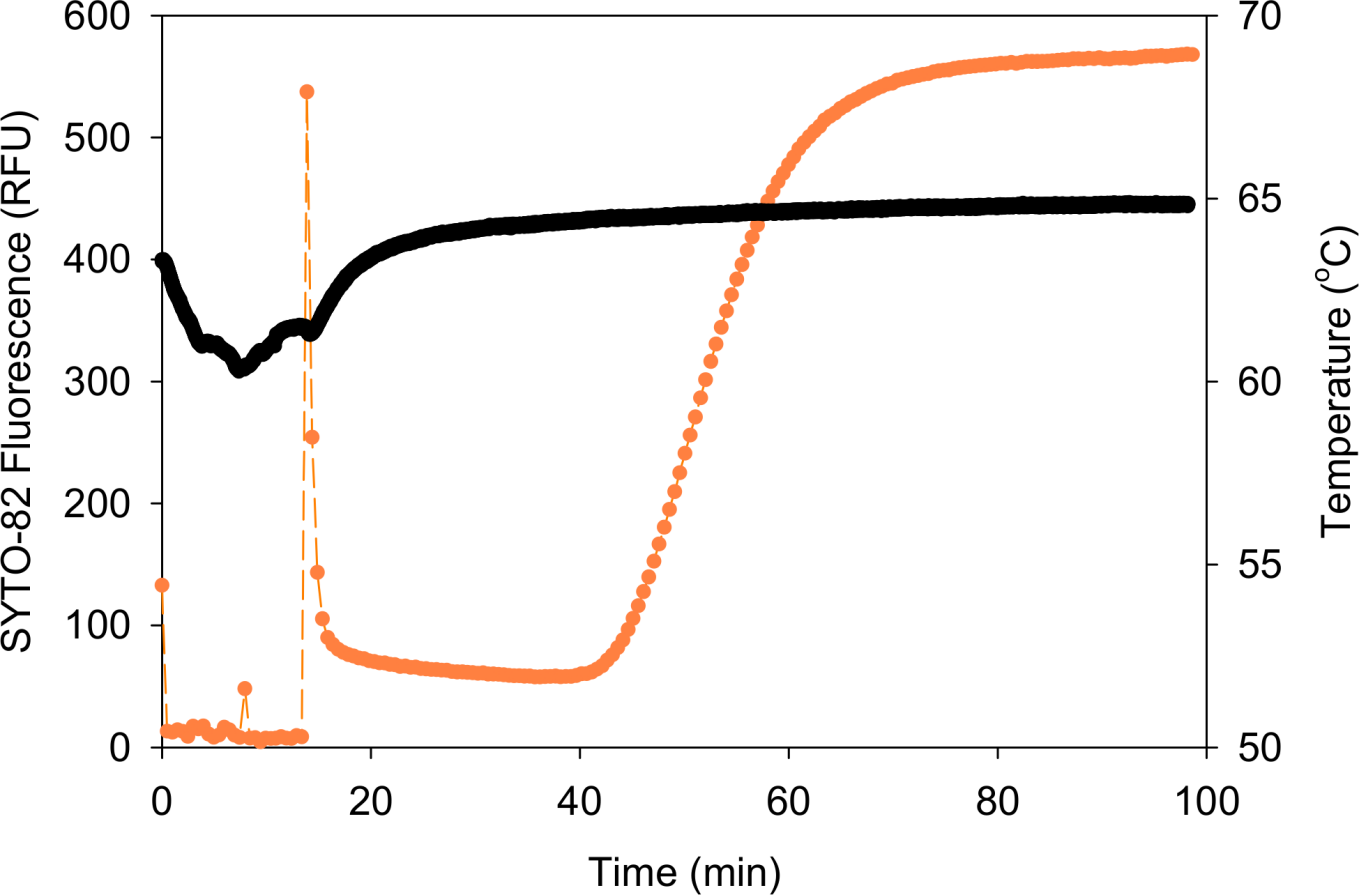
Example raw data from integrated extraction and amplification assay. Fluorescence (orange) and temperature (black) were recorded during extraction of TB DNA from a high concentration PATH surrogate sputum sample and LAMP amplification. During the extraction phase of the assay, the temperature was transiently reduced by approximately 5°C as the room temperature tubing passed through the block. The peak in fluorescence at approximately 15 minutes indicated that the reaction chamber of the tubing was positioned correctly in the heat block as the amplification phase of the assay began.

Using the automated, integrated device, TB DNA was extracted from surrogate sputum samples and amplified by LAMP. The heat block in the device was brought to 65°C before the assay was started. During the 15 minute extraction phase of the assay, the temperature was transiently reduced by approximately 5°C as the room temperature tubing passed through the heat block (Fig 6, black). Small peaks in fluorescence were seen as the reaction chamber of the tube passed in front of the fluorescence reader during the sample preparation phase of the assay (Fig 6, orange). Once DNA extraction was completed, a characteristic peak in fluorescence was recorded at approximately 15 minutes indicating that the reaction chamber was correctly positioned in the heat block (Fig 6, orange). As the temperature in the tube increased from room temperature to 65°C, the fluorescence of the SYTO-82 intercalating dye decreased as the nonspecific DNA interaction present in the sample at room temperature was reduced. After approximately 30 min, the fluorescence curve characteristic of DNA amplification was observed.

**Fig 7:**
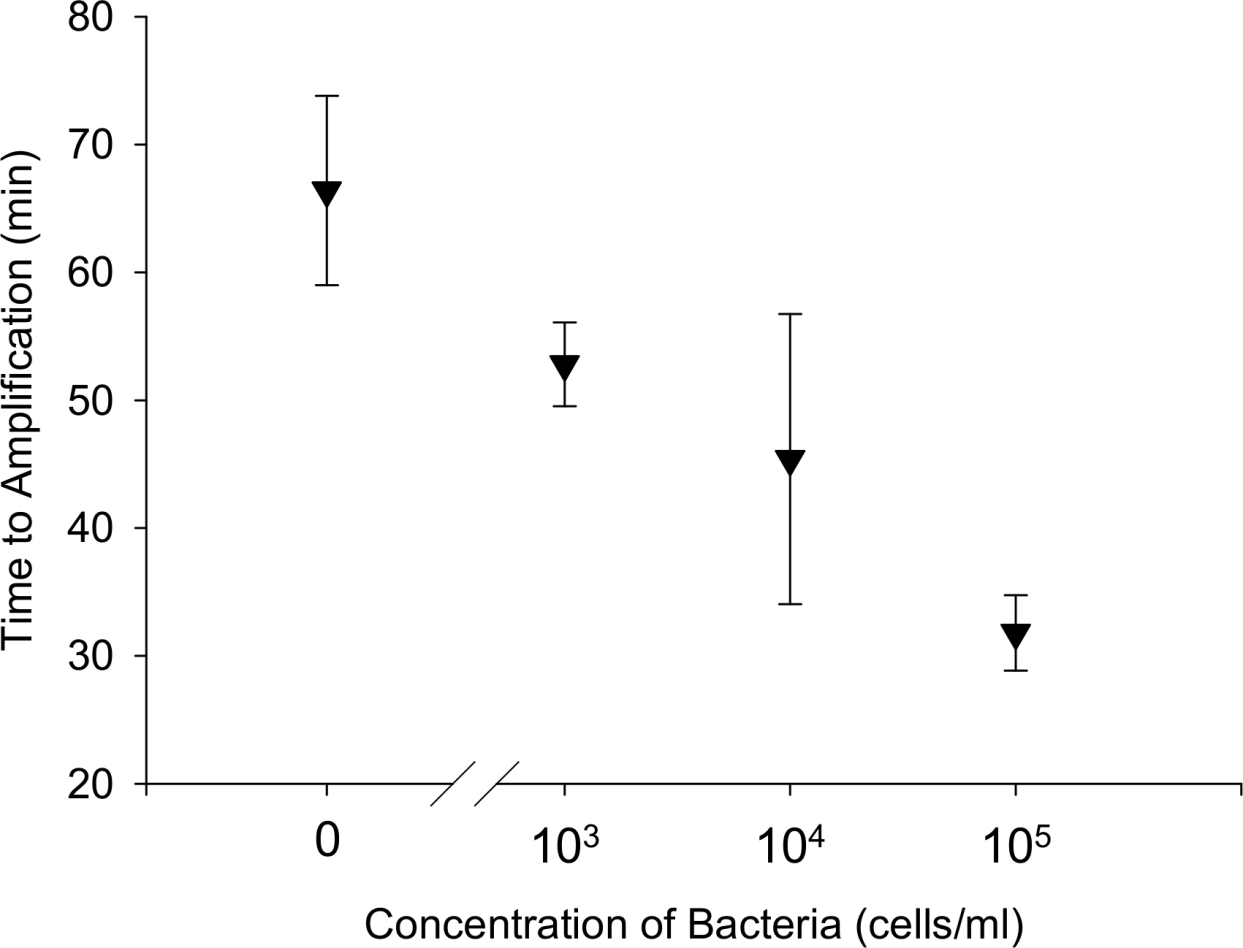
LAMP amplification of TB DNA from surrogate sputum samples. Time to amplification for lysed surrogate sputum samples in integrated extraction/amplification device; N = 5.

LAMP amplification of all three TB concentrations was seen (Fig 7) using the integrated extraction and amplification device (Fig 2). The low samples (10^3^ cells/mL) amplified at 52.8 ± 3.3 minutes, the medium samples (10^4^ cells/mL) at 45.4 ± 11.3 minutes, and the high samples (10^5^ cells/mL) at 31.8 ± 2.9 minutes (N = 5). Surrogate sputum samples without TB bacteria amplified at 66.4 ± 7.4 minutes (N = 5), statistically longer than the three concentrations of TB sample (Fig 7).

## Discussion

This study demonstrates the feasibility of combining TB DNA extraction from sputum samples with isothermal amplification and detection of the IS6110 gene within a single section of tubing. When DNA from lysed surrogate sputum samples was pulled through the extraction tubing into a LAMP reaction solution, the DNA eluted from the beads and amplified, as shown by characteristic exponential amplification curves (Fig 6). Amplification was seen with LAMP for all three concentrations of TB in surrogate sputum (Fig 7), and all three concentrations had significantly shorter amplification times than the negative control samples.

Design of the integrated, automated assay required means to move the magnetic beads through the extraction solutions into the reaction solution, to heat the isothermal reaction, and to read the fluorescence of the reaction. In the original sample preparation technique, a single external magnet was manually moved along the length of the tubing, varying speed and distance from the tube to achieve mixing of the beads within each chamber and movement between the chambers [14–17]. In the automated design, two attracting magnets were fixed, and the tubing was moved between the magnets using positioning rollers (Fig 2). We found that with sputum samples and a single fixed magnet, the beads were compressed on the wall of the tubing at the edge of the magnet and, as a consequence, were poorly dispersed in the next solution. This was improved by using two attracting magnets to establish magnetic gradients that position the beads towards the center of the tube and the center of the magnets. The two attracting magnets design moved the beads through the tubing with less sample compression and better bead dispersion, improving biomarker extraction.

Extraction of DNA was followed by amplification within the extraction tubing by means of a Peltier heater and detected with a fluorescence reader (Fig 2). In the integrated manual version of this design, after the DNA was eluted into the reaction solution the tubing was mounted vertically with the reaction chamber in contact with the Peltier heater. Temperature was monitored by a thermal camera, and fluorescence was recorded by an ESEQlog fluorescence detector. In the automated design, a copper heat block in contact with the Peltier heater was used to provide more uniform heating of the tubing and reaction solution. The thermal camera was replaced by a witness tube containing mineral oil and a thermocouple for recording and monitoring temperature. The ESElog fluorescence detector was mounted at a right angle to the heat block and aligned with an opening to allow optical monitoring of the isothermal reaction.

The final integrated design was still not fully automated. Temperature was set manually before each experiment. In a laboratory environment, this temperature setting did not change significantly between experiments, but feedback to control the block temperature would reduce user intervention. Similarly, the simple program that controlled the tube positioning motor and the fluorescence reader simultaneously was triggered at the start of the experiment but ran autonomously. As a result, fluorescence data was collected during the sample preparation phase of the assay. Temperature and fluorescence data were time stamped to allow data alignment and to confirm the start time of the amplification assay (Fig 6). More sophisticated automated designs that integrate temperature control, optical measurement, and motion control to optimize data collection are currently in development.

A chemical lysis method and extraction technique were coupled with isothermal amplification in an integrated device to detect TB bacteria in surrogate sputum samples. As a TB diagnostic, the DNA extraction and amplification reagents would be pre-arrayed in the tubing requiring only the insertion of the lysed patient sample by the technician. The extraction technology has been employed with blood and urine samples [15,17], and the technology incorporating LAMP amplification could also be adapted to other patient sample matrices. In the future, detection of amplification product with a fluorescent probe instead of an intercalating dye would likely decrease false positive assays and possibly increase sensitivity. A TB probe would also allow the addition of an extraction control that could be included in the lysis/binding buffer, extracted with the target DNA, and detected with a second optical probe. This study demonstrated the feasibility of integrating sample preparation and isothermal amplification in a self-contained system which, with further development, could potentially be useful for point-of-care applications in a low-resource setting.

## Supporting Information

S1 Fig Tubing movement map. (PDF)
