# Supplementary material for: Tuberculosis Biomarker Extraction and Isothermal Amplification in an Integrated Diagnostic Device
Source: PLoS One. 2015 Jul 1;10(7):e0130260. doi: 10.1371/journal.pone.0130260 (PMC4488445; doi:10.1371/journal.pone.0130260)

Tubing Movement Map

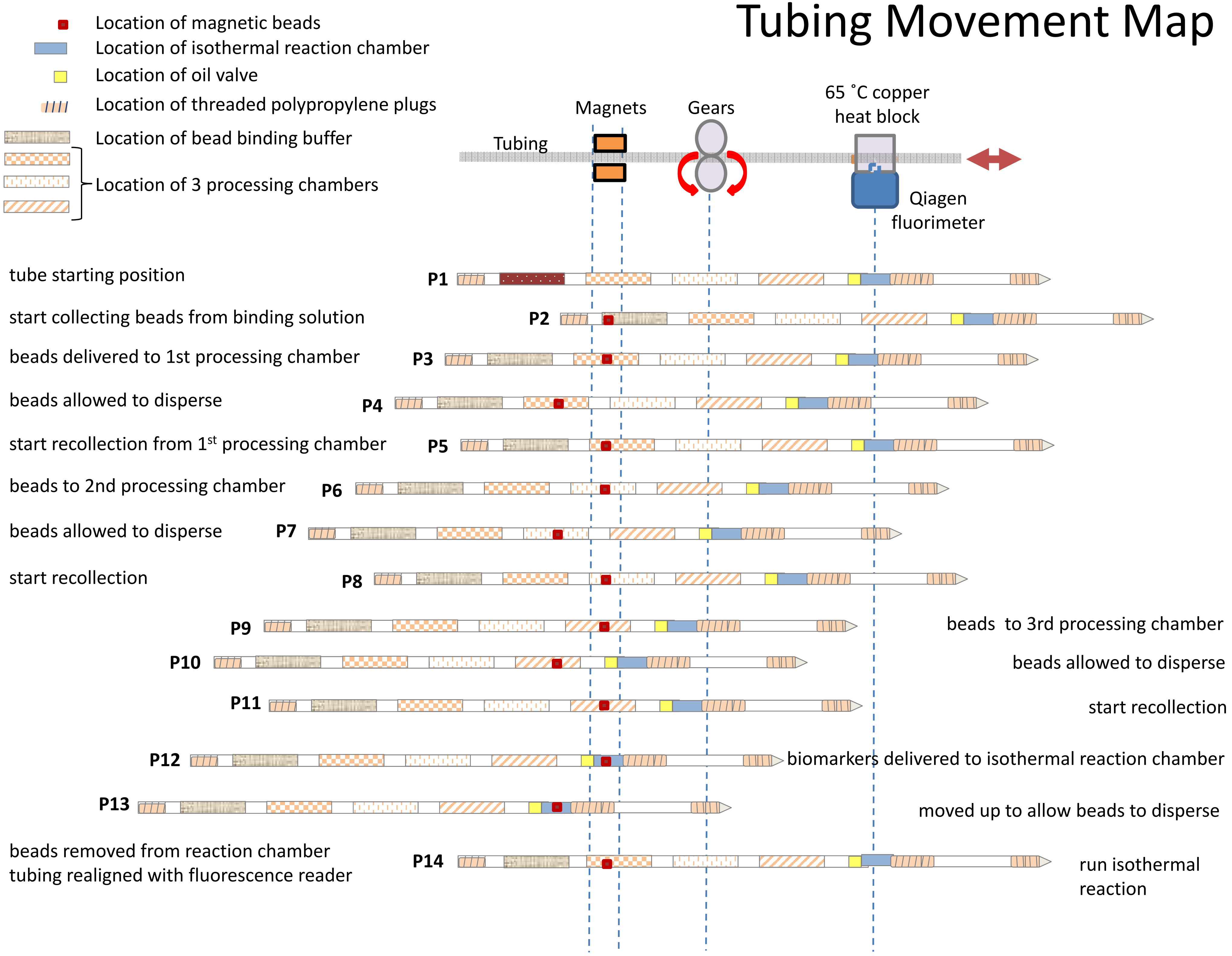

Supplement: S1 Fig — (PDF) [file pone.0130260.s001.pdf]
